# Improved Open- Circuit Voltage in ZnO–PbSe Quantum Dot Solar Cells by Understanding and Reducing Losses Arising from the ZnO Conduction Band Tail

**DOI:** 10.1002/aenm.201301544

**Published:** 2014-02-21

**Authors:** Robert L Z Hoye, Bruno Ehrler, Marcus L Böhm, David Muñoz-Rojas, Rashid M Altamimi, Ahmed Y Alyamani, Yana Vaynzof, Aditya Sadhanala, Giorgio Ercolano, Neil C Greenham, Richard H Friend, Judith L MacManus-Driscoll, Kevin P Musselman

**Affiliations:** Department of Materials Science and Metallurgy, 27 Charles Babbage Road, University of CambridgeCambridge, CB3 0FS, UK; Department of Physics, JJ Thomson Avenue, University of CambridgeCambridge, CB3 0HE, UK; Instituto de Ciencia de Materiales de Barcelona, ICMAB-CSIC, Campus de la UABBellaterra, 08193, Spain; Petrochemicals Research Institute, King Abdulaziz City for Science and TechnologyRiyadh, 11442, Kingdom of Saudi Arabia; National Nanotechnology Research Centre, King Abdulaziz City for Science and TechnologyRiyadh, 11442, Kingdom of Saudi Arabia

## Abstract

Colloidal quantum dot solar cells (CQDSCs) are attracting growing attention owing to significant improvements in efficiency. However, even the best depleted-heterojunction CQDSCs currently display open-circuit voltages (*V*_OC_s) at least 0.5 V below the voltage corresponding to the bandgap. We find that the tail of states in the conduction band of the metal oxide layer can limit the achievable device efficiency. By continuously tuning the zinc oxide conduction band position via magnesium doping, we probe this critical loss pathway in ZnO–PbSe CQDSCs and optimize the energetic position of the tail of states, thereby increasing both the *V*_OC_ (from 408 mV to 608 mV) and the device efficiency.

## 1. Introduction

Depleted-heterojunction colloidal quantum dot solar cells (CQDSCs) have attracted significant attention because they have yielded the most efficient quantum dot solar cells, whilst the materials (metal oxide and quantum dot films) are low-cost, abundant, and solution processable.[1–6] In addition to the commercial appeal, the bandgap (*E_g_*) of the QDs can be widely tuned over the visible spectrum to optimize light absorption.[3,7] However, the open-circuit voltages (*V*_OC_s) of these devices are, to date, approximately 0.5–0.8 V below *E_g_*/*q*, where *q* is the electron charge, even in the most efficient cases.[1,8] Fundamentally, higher *V*_OC_s (around 0.3–0.4 V below *E*_g_/*q* for bandgaps of 1.5 eV or below) should be possible based on Shockley–Queisser considerations.[9,10] Voltage losses in the quantum dot film and at interfaces in these devices have been studied in some detail and addressed via advanced synthetic methods,[3,11,12] but the metal oxide may also be responsible for the energetic losses.[5] In this work, we continuously raise the conduction band position of the zinc oxide in ZnO–PbSe CQDSCs by doping with magnesium. Our study indicates that the tail of ZnO conduction band states fundamentally limits the efficiency of CQDSCs to a value less than the ideal. However, by tuning the oxide's conduction band position, we are able to minimize this loss and significantly improve the *V*_OC_ to achieve the highest value reported for ZnO–PbSe CQDSCs.

## 2. Results and Discussion

**Figure 1:**
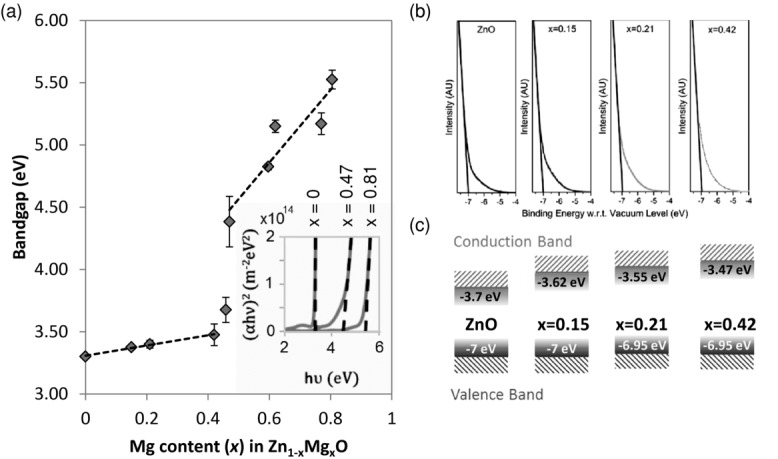
a) Bandgap (*E*_g_) vs. Mg content (*x*) of Zn_1–x_Mg_x_O deposited under optimized conditions with Tauc plots inset. b) Valence band positions for ZnO through to Zn_0.58_Mg_0.42_O measured using UPS. The difference between −7 eV and −6.95 eV is within the error of the instrument, indicating that the valence band position was unchanged between *x* = 0 and *x* = 0.42. c) Valence band and conduction band positions of the Zn_1–x_Mg_x_O with shaded band tails illustrated. The conduction band positions were calculated using the UPS data and bandgap measurements.

For the purpose of this study, Mg-doped ZnO films (Zn_1–x_Mg_x_O) were deposited using the low-temperature atmospheric (or spatial) atomic layer deposition (AALD) process, which is rapid and roll-to-roll compatible.[13–16] In AALD, the substrate is oscillated <100 μm beneath different gas channels (gaseous metal precursors, inert N_2_ gas, and oxidant gas) to replicate the conventional ALD cycles in open-atmosphere. However, available Mg precursors have very low vapor pressures,[17,18] such that introducing sufficient Mg precursor into the gas phase (by bubbling with nitrogen) is challenging and the synthesis of Zn_1–x_Mg_x_O has not been previously reported by any spatial ALD technique. By carefully controlling the bubbling rates and heating the Mg precursor, we were able to produce Zn_1–x_Mg_x_O by AALD and continuously vary its bandgap from 3.3 eV (*x* = 0) up to 5.5 eV (*x* = 0.81), as demonstrated in **Figure** 1a (see Section S1 of the Supporting Information for experimental details). These bandgaps were determined from the corresponding Tauc plots (inset in Figure 1a).[19] The bandgap increased gradually at first, then sharply for *x* > 0.42 due to the formation of the rocksalt Mg_x_Zn_1–x_O phase, which has a higher coordination number (6 cf. 4) than the wurtzite Mg-doped ZnO phase (Supporting Information, Section S1).[20,21] Ultraviolet photoelectron spectroscopy (UPS) measurements indicated that the position of the valence band was unchanged for Mg doping levels up to *x* = 0.42 (Figure 1b), suggesting that the bandgap increase resulted from a shift in the conduction band position (Figure 1c). Notably, our technique allowed very reproducible bandgap tuning, as indicated by the small error bars in Figure 1a.

A tail of states extending into the bandgap was observed for our Zn_1–x_Mg_x_O. The onset of absorption in the Tauc plots (inset of Figure 1a) and the onset of photoemission in the UPS plots (Figure 1b) both showed the presence of a tail of states. These observations are in agreement with those made on ZnO and TiO_2_ sol-gel films,[5,22–24] which are commonly used in CQDSCs.[25] Band tails arise from disorder, which may be due to impurities, defects at grain boundaries, or the interaction of excitations with the lattice.[10,26–29] For comparison, polycrystalline Zn_1–x_Mg_x_O films were also produced in this work by pulsed laser deposition (PLD) (in vacuum at 450 °C) and likewise showed the presence of a band tail (Section S2 of the Supporting Information). This emphasizes that these tail states are not exclusive to materials synthesized in open-atmosphere using low-temperatures processes, but instead can follow from the polycrystalline nature of the metal oxide films. The characteristic feature of a conduction band tail is an exponential decay in the density of states below the conduction band minimum.[26,27] By doping ZnO with magnesium to vary the position of its conduction band and density of states, we were able to study the effect of this tail of states on the performance of CQDSCs.

**Figure 2:**
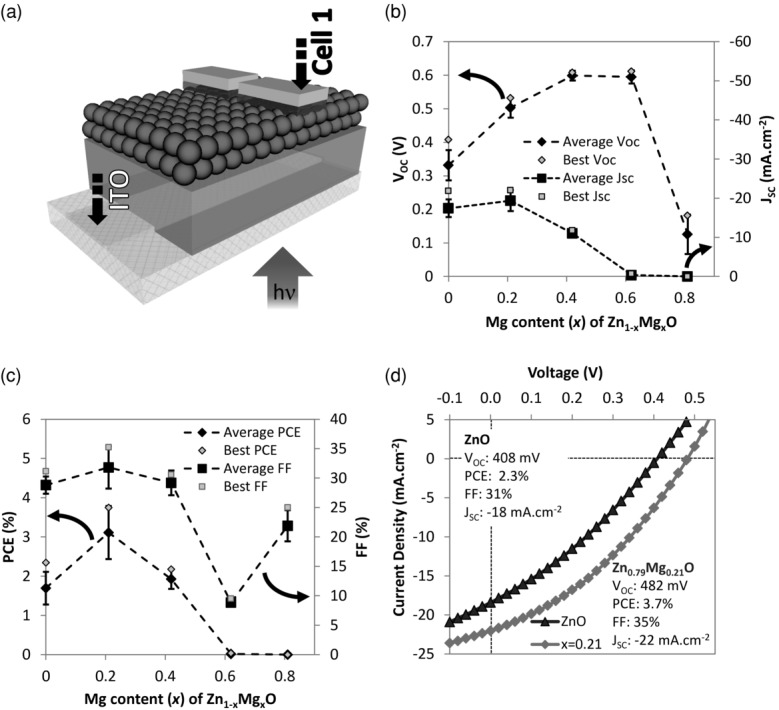
a) Device structure b) *V*_OC_ and *J*_SC_, c) *PCE* and *FF* of the Zn_1–x_Mg_x_O–PbSe CQDSCs over a doping series measured under 1-sun AM 1.5G illumination. d) Light *J*–*V* curves comparing the highest efficiency Zn_1–x_Mg_x_O-PbSe CQDSC with the most efficient ZnO-PbSe CQDSC from this work.

Bilayer CQDSCs, schematically drawn in **Figure** 2a, consisted of 200 nm thick Zn_1–x_Mg_x_O films covered with 1.43 eV bandgap PbSe QDs, which were capped with MoO_3_/Au contacts. The device synthesis method is similar to previous reports and the details are described in the Experimental Section.[7] Upon Mg-doping of the ZnO layer, the *V_OC_* increased from 330 ± 50 mV (highest value 408 mV) for undoped ZnO up to 600 ± 10 mV (highest value 608 mV) for Zn_0.58_Mg_0.42_O (Figure 2b). A *V_OC_* of 608 mV is, to our knowledge, the highest reported for ZnO–PbSe CQDSCs.[1,3,4,25,30,31] Figure 2b and 2c also show that the phase transition from wurtzite Zn_x_Mg_1–x_O to the more insulating rocksalt Mg_x_Zn_1–x_O phase at 42 at% Mg content (Supporting Information, Figure S1b) resulted in a significant reduction in the short-circuit current density (*J_SC_*) and power conversion efficiency (*PCE*). Hence, the study of the ZnO conduction band tail was focused on compositions where there was only the wurtzite phase.

**Figure 3:**
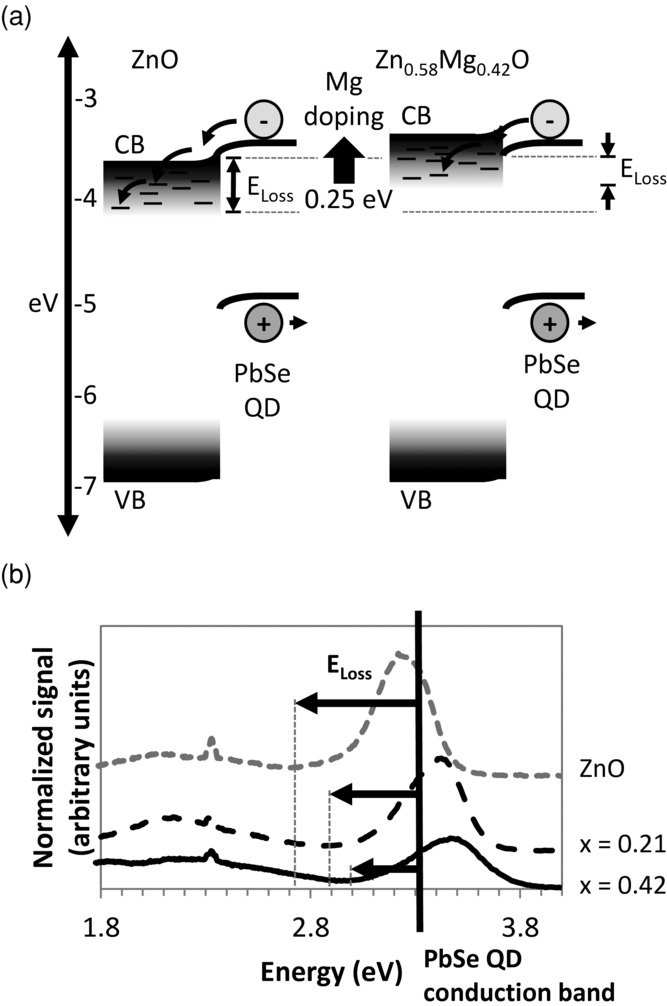
a) Illustration of the thermalization of electrons transferred from the PbSe QDs to the Zn_1–x_Mg_x_O in devices with *x* = 0 (left) and *x* = 0.42 (right). The sub-bandgap states in the Zn_1–x_Mg_x_O are drawn such that darker shading indicates a higher density of states (as suggested by the intensity of the photoluminescence measurements). b) Photoluminescence spectra of Zn_1–x_Mg_x_O indicating a shift in the tail of sub-bandgap states beneath the Zn_1–x_Mg_x_O conduction band to higher energies with Mg doping. The dashed lines indicate the lowest energy emissive state (which was estimated from the minimum PL intensity below the excitonic peak) and the bold line illustrates the approximate position of the PbSe conduction band (relative to the Zn_1–x_Mg_x_O valence band), which together provide an upper estimate of the energy loss due to thermalization.

The conduction band position of the undoped ZnO was measured to be −3.7 eV (Figure 1c). This is similar to the conduction band level of the PbSe quantum dots (−3.67 eV, see Supporting Information, Figure S4). The band diagram for the device with undoped ZnO is shown on the left of **Figure** 3a, where we illustrate the tails in the density of band states extending into the bandgap of the Zn_1–x_Mg_x_O. Photoluminescence measurements further suggested that band tails were present in our films. The band emission for undoped ZnO was observed to extend ∽0.5 eV below the optical gap (Figure 3b). In the presence of a conduction band tail, electrons transferred from the QDs to the ZnO may thermalize down the tail of sub-bandgap states to lower energy levels in the ZnO or they may be transferred to lower energy states in the ZnO directly at the interface. Either situation corresponds to a reduction in the extracted energy and achievable *V*_OC_, in a manner similar to that observed previously in light-absorbing layers with tail states in silicon and organic solar cells.[10]

With Mg doping, the oxide conduction band shifts to higher energies, as shown on the right of Figure 3a. Fewer Zn_1–x_Mg_x_O sub-bandgap states are then expected to be available beneath the PbSe QD conduction band. This reduces the subsequent energy loss and hence increases the *V*_OC_. This idea is strongly supported by the photoluminescence results (Figure 3b), where it is seen that the lowest energy emissive band-edge states in the oxide shift to higher energy levels with doping, likely bringing them closer to the PbSe conduction band. Absorption measurements (Supporting Information, Figure S5) likewise indicate a shift in the tail of sub-bandgap states with doping, leading to a reduction in the energy loss in the devices. We note that the intensity of the excitonic peak in the photoluminescence measurements of the metal oxides (Figure 3b) decreased with increasing doping, indicating an increasing density of defect states. However, absorption measurements show that the increase was mainly in the density of mid-bandgap states, rather than the band-tail (Figure S5).

**Figure 4:**
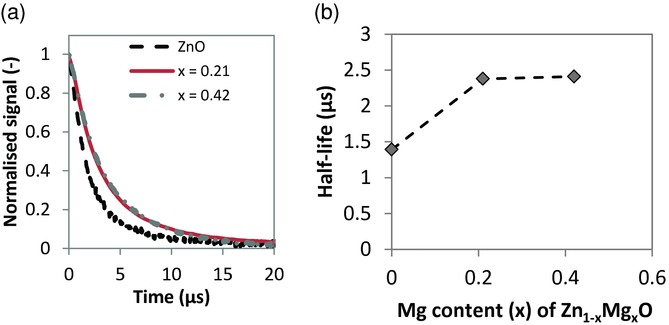
Transient photovoltage measurements of Zn_1–x_Mg_x_O–PbSe CQDSCs. a) Plot of normalized open-circuit voltage perturbation against time and b) plot of open-circuit voltage decay half-life against magnesium content (*x*). The measurements were performed under 1-sun background illumination.

It is likely that a reduction in the interfacial recombination with Mg doping also contributed to the improved *V*_OC_.[32] We have previously shown that reducing the ZnO carrier concentration through nitrogen doping can limit interfacial recombination in CQDSCs,[3] and we observed a reduction in the ZnO carrier concentration here with Mg doping (Supporting Information, Figure S6). Transient photovoltage measurements performed under 1-sun background illumination indicated a small increase in the half-life of the voltage perturbation from *x* = 0 to a Mg content of *x* = 0.21, consistent with slower interfacial recombination (**Figure** 4). However, there was almost no increase in the photovoltage half-life when the Mg content was varied from *x* = 0.21 to *x* = 0.42 (Figure 4), whereas the *V*_OC_ increased by approximately a further 100 mV (Figure 2b). Furthermore, in our previous work with nitrogen-doped ZnO, the *V_OC_* of ZnO:N–PbX (X = PbSe, PbS) CQDSCs only increased by 180 mV for a two order of magnitude decrease in the carrier concentration,[3] whereas here we achieved a larger *V*_OC_ change when the carrier concentration decreased by only one order of magnitude from *x* = 0 to a Mg content of *x* = 0.6. Notably, from *x* = 0 to *x* = 0.42, the *V*_OC_ increased by 270 mV, which closely corresponded with the bandgap shift of 250 meV. These considerations indicate that the optimization of the Zn_1–x_Mg_x_O conduction band position, rather than reduced interfacial recombination, was the major reason for the increased *V*_OC_.

The solar cell parameters in Figure 2b show that there was a compromise between increased *V_OC_* and reduced *J_SC_* with increasing Mg doping. A measurable photocurrent was still obtained when the Zn_1–x_Mg_x_O conduction band was above that of the PbSe QDs (when *x* > 0), indicating that a tail of conduction band states in the Zn_1–x_Mg_x_O was actively accepting electrons from the PbSe quantum dots and transporting electrons to the electrode. However, the density of accessible tail states is expected to decrease exponentially with doping as the conduction band was shifted to higher energies (as shown in the right of Figure 3a), in agreement with the decrease in *J_SC_* with increasing Mg doping.

**Figure 5:**
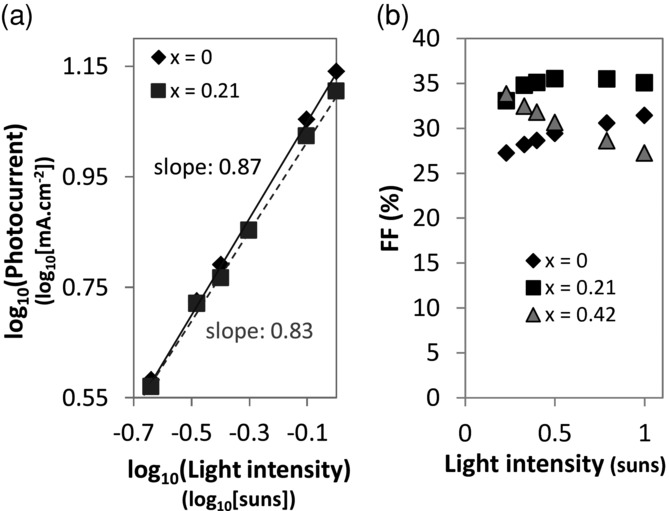
a) Photocurrent for varying light intensity (1 sun = 100 mW.cm^−2^ under AM 1.5G illumination) at *V*_0_–*V* = 0.35 V (see Supporting Information Figure S7) such that the slope gives the power of the relationship between the photocurrent and light intensity. b) Fill factor vs. light intensity (suns).

If the reduction in *J_SC_* at higher magnesium doping was due to a limited number of accepting states in the tail of the Zn_1–x_Mg_x_O conduction band, the accumulation of photogenerated electrons in the PbSe at the heterojunction would be expected. Following the analysis developed by Goodman and Rose,[33–35] we verified this by measuring the photocurrent (light current minus the dark current) against the effective applied voltage *V_0_–V* (where *V_0_* is the bias at which the photocurrent is zero) and identifying the region with a square-root relationship between the photocurrent and the effective applied voltage. If the photocurrent follows the light intensity to the power three-quarters in that region, then the solar cell photocurrent is space-charge limited.[3,33–35] We found that the photocurrent of a device with undoped ZnO showed a power of 0.87, but when doped (*x* = 0.21), this value decreased to 0.83, indicating that the device was approaching a space-charge limited regime (see **Figure** 5a and Figure S7 in the Supporting Information). We also found that the Zn_1–x_Mg_x_O–PbSe devices with higher magnesium contents had higher performance at low light intensities than high light intensities, as indicated by the slope of the fill factor (*FF*) vs. light intensity (Figure 5b) and white-light biased EQE measurements (Supporting Information, Figure S8). These studies agree with the assertion that when the Zn_1–x_Mg_x_O conduction band is raised sufficiently such that only a small density of tail states are able to accept electrons from the PbSe, charge generation in the PbSe kinetically outcompetes charge transfer to the Zn_1–x_Mg_x_O such that space-charge accumulates in the device under illumination and increases the amount of recombination.

The best compromise between *V*_OC_ and *J*_SC_ was found for *x* = 0.21 (Figure 2c), where the efficiency was increased to 3.7% from 2.3% for *x* = 0 (see Figure 2d for *J*–*V* curves). A 3.7% efficiency is comparable with the best in the literature for ZnO–PbSe CQDSCs,[1,4,25,31] and is notable considering that the Zn_1-x_Mg_x_O was produced by a scalable, low temperature technique compatible with printing on plastic substrates.[13,15,16] The open-circuit voltages and efficiencies of these devices based on AALD Zn_1–x_Mg_x_O exceeded those of identical devices made using the PLD Zn_1–x_Mg_x_O, despite the fact that the PLD films were deposited in vacuum at a higher temperature (450 °C) (Supporting Information, Section S2). We attribute this in part to better formation of the PbSe QD layer on the smoother AALD Zn_1–x_Mg_x_O films. Despite these merits, the trade-off between *V*_OC_ and *J*_SC_ illustrates a fundamental loss mechanism associated with the presence of a tail in the density of conduction band states in the metal oxide. When the conduction band of zinc oxide is raised, more energy can be extracted per electron because the thermalization of electrons to lower-energy tail states is reduced. However, due to the exponential decay of the density of states in the conduction band tail, fewer acceptor states are available in the zinc oxide when the band is raised, resulting in a reduction in photocurrent. Our results suggest that even higher efficiencies will be attainable using acceptors without a pronounced tail in their density of conduction band states, providing motivation for the development of alternative acceptor materials with cleaner bandgaps. Organic semiconductors[7] and complementary quantum dot layers[36] are promising alternatives in this regard, but may not afford the same advantages as metal oxides in terms of stability and ease of processing.

## 3. Conclusion

In conclusion, we observed a tail of defect states extending into the bandgap of zinc oxide, characteristic of polycrystalline films, and found that these states reduce the energy of electrons collected from the ZnO–PbSe CQDSCs and hence the achievable *V*_OC_. By continuously raising the conduction band tail of ZnO through Mg doping to reduce this loss, we were able to increase the *V*_OC_ from 408 mV to 608 mV, the highest reported to date for ZnO–PbSe CQDSCs. Notably, we achieved this using a scalable AALD technique that is suitable for the commercialization of CQDSCs. But we found that the small density of acceptor states near the bottom of the tail limits the useful Mg doping range, such that the loss in *V*_OC_ induced by the tail of states cannot be entirely recovered. The ability to precisely tune the conduction band position of the Zn_1–x_Mg_x_O allowed us to identify an optimal position for the oxide conduction band and its associated tail of states, which resulted in an increase in the power conversion efficiency from 2.3% to 3.7%. This work emphasizes the strong influence that the metal oxide properties can have on device performance. It also identifies a fundamental loss pathway in polycrystalline metal oxide films, as well as providing important direction for the development of electron-acceptor materials for CQDSCs.

## 4. Experimental Section

*AALD Film Preparation*: The ITO/glass substrates used (Colorado Concept Coatings LLC) were washed in soapy water and ultrasonically cleaned in acetone and isopropanol. The Zn_1–x_Mg_x_O deposition onto the substrates was similar to a previously reported method.[13] The main difference was the rates at which nitrogen gas was bubbled through the precursors: H_2_O (100 mL min^−1^), diethylzinc (0.8–10 mL min^−1^), and Mg(CpEt)_2_ (180–500 mL min^−1^). These are detailed in Table S1 in the Supporting Information.

*Quantum Dot Synthesis*: The PbSe QD synthesis and device fabrication were according to a previously reported method.[7] For the synthesis, the key difference was that the Se solution in tri-*n*-octylphosphine was injected into the Pb-oleate precursor at 125 °C. After 6 s the reaction was quenched by adding cold hexane (−20 °C) and placing the flask into an ice water bath. The QDs were then purified by repeated flocculation using the solvent-nonsolvent mixture of hexane and butanol/methanol in an argon filled glove box. The QDs were stored in octane under an argon atmosphere. For the device fabrication, the notable difference was that we used five iterations of the layer-by-layer procedure to deposit the nanocrystals from solution (25 mg mL^−1^), resulting in approximately 100 nm thick films. As the top contact, MoO_3_ and Au were deposited by thermal evaporation.

*Film and Device Characterization*: The bandgap of the Zn_1–x_Mg_x_O films was measured using an Agilent/HP 8453 UV-visible spectrometer. X-ray diffraction patterns were obtained using a Bruker D8 theta/theta system using CuK_α_ radiation (1.5406 Å wavelength). Simulated AM1.5G solar illumination was provided using a Newport Oriel Class A solar simulator, at an intensity equivalent to 100 mW cm^−2^ after correcting for spectral mismatch. Three to six devices were characterized for each metal oxide doping level, and their photovoltaic performances averaged. The contact area was 4.5 mm^2^, although the effective device area may be slightly larger due to current collection from the area surrounding the electrode. This correction is expected to be small and was not addressed here, given our interest in performance trends rather than the absolute value of current density. Photoluminescence spectra were measured at room temperature using an ACCENT RPM 2000 system with a Nd:YAG laser (266 nm wavelength, 4.5 mW power). Photoelectron spectroscopy (UPS/XPS) was performed in an ultrahigh-vacuum chamber (ESCALAB 250Xi). For UPS measurements a double-differentially pumped He gas discharge lamp emitting He I radiation (*hν* = 21.22 eV) with a pass energy of 2 eV was used. XPS measurements were carried out using an XR6 monochromated Al KR X-ray source (*hν* = 1486.6 eV) with a 900 μm spot size to determine the amount of Mg doping in the oxide films. The transient photovoltage measurements were obtained with a 1 MΩ impedance across the terminals of the cell measured. A 550 nm wavelength diode was pulsed with a width of 50 ms on the cell to ensure that the light perturbation caused the voltage across the cell to reach saturation. The voltage vs. time was recorded and the voltage decay normalized and fitted with an exponential expression to determine the half-life of the decay.
