# Supplementary material for: Improved Open- Circuit Voltage in ZnO–PbSe Quantum Dot Solar Cells by Understanding and Reducing Losses Arising from the ZnO Conduction Band Tail
Source: Adv Energy Mater. 2014 Feb 21;4(8):1301544. doi: 10.1002/aenm.201301544 (PMC4511390; doi:10.1002/aenm.201301544)
Supplement: Supplementary file 1 [file aenm0004-0001-sd1.pdf]

Copyright WILEY-VCH Verlag GmbH & Co. KGaA, 69469 Weinheim, Germany, 2014.

ADVANCED ENERGY MATERIALS

Supporting Information

for *Adv. Energy Mater.,* DOI: 10.1002/aenm.201301544

Improved Open–Circuit Voltage in ZnO–PbSe Quantum Dot Solar Cells by Understanding and Reducing Losses Arising from the ZnO Conduction Band Tail

*Robert L. Z. Hoye, Bruno Ehrler, Marcus L. Böhm, David Muñoz-Rojas, Rashid M. Altamimi, Ahmed Y. Alyamani, Yana Vaynzof, Aditya Sadhanala, Giorgio Ercolano, Neil C. Greenham, Richard H. Friend, Judith L. MacManus-Driscoll, and Kevin P. Musselman**

Copyright WILEY-VCH Verlag GmbH & Co. KGaA, 69469 Weinheim, Germany, 2013.

Supporting Information

for *Adv. Energy Mater.*, DOI: 10.1002/aenm.201301544

Improved Open – Circuit Voltage in ZnO – PbSe Quantum Dot Solar Cells by Understanding and Reducing Losses Arising from the ZnO Conduction Band Tail

*Robert L.Z. Hoye, Bruno Ehrler, Marcus L. Böhm, David Muñoz-Rojas, Rashid M. Altamimi, Ahmed Y. Alyamani, Yana Vaynzof, Aditya Sadhanala, Giorgio Ercolano, Neil C. Greenham, Richard H. Friend, Judith L. MacManus-Driscoll, and Kevin P. Musselman**

Section S1. Optimized Conditions for Synthesizing $Zn_{1-x}Mg_xO$ by Atmospheric ALD

Table S1. Deposition conditions of AALD $Zn_{1-x}Mg_xO$ films and their compositions determined from XPS. $Mg(CpEt)_2$ is the Mg precursor (bis(ethylcyclopentadienyl)magnesium) and DEZ is the Zn precursor (diethylzinc).

Bubbling rate through precursor (mL/min)		Mg(CpEt)₂/DEZ flow rate ratio	*x*
$Mg(CpEt)_2$	DEZ		
0	10	0	0
200	8	25	0.15
200	6	33	0.21
180	4	45	0.42
240	4	60	0.46
500	6.7	75	0.47
500	5.7	88	0.60
500	4.00	125	0.62
500	1.67	300	0.77
500	0.80	625	0.81

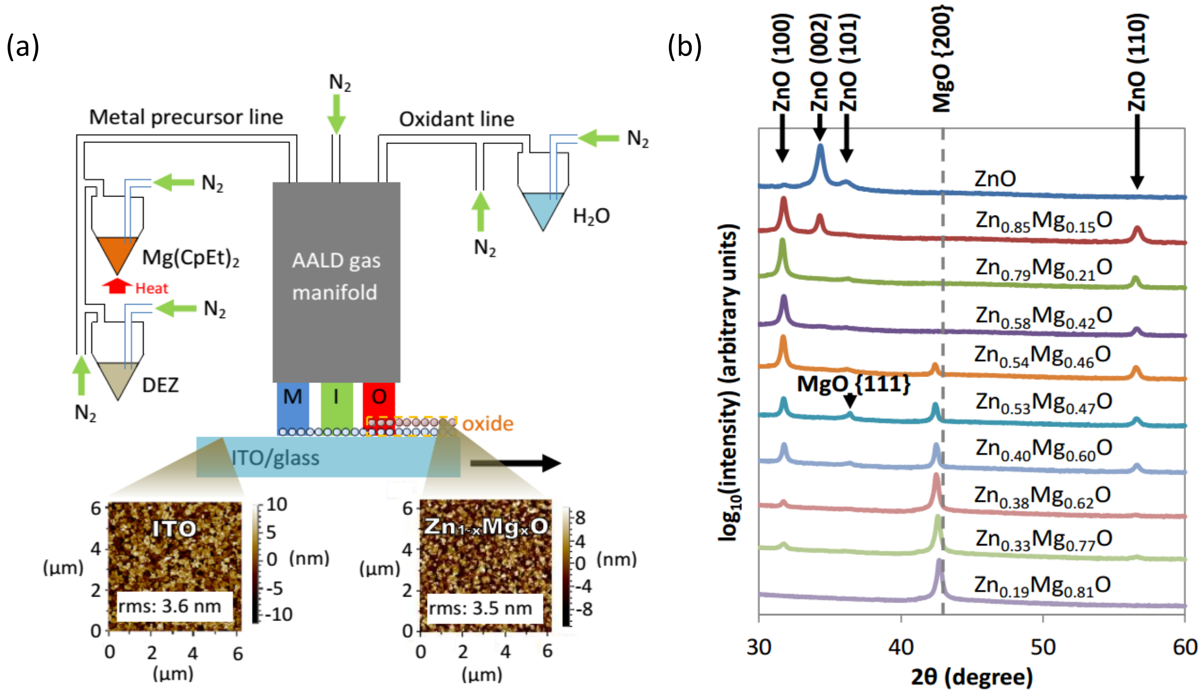

Figure S1. (a) Simplified diagram of the AALD gas manifold showing how a monolayer of oxide can be deposited onto the ITO/glass substrate in one pass using our close-proximity Atmospheric Atomic Layer Deposition (AALD) system.[1,2] Afterwards, the substrate moves back through the gas channels in the opposite direction, and so forth to obtain the desired film thickness. The precursors were introduced to the gas phase by bubbling carrier gas (N_2)

through the liquid precursors. The AFM images, obtained using an Agilent 5500 SPM in Acoustic AC Mode, show that the deposited $Zn_{1-x}Mg_xO$ film was conformal to the ITO substrate. (b) X-ray diffraction patterns. The Mg content was determined from XPS measurements. For $x > 0.42$, the presence of rocksalt $Mg_xZn_{1-x}O$ alloy rather than MgO is indicated by the significant displacement of the MgO {200} peaks from the literature value of 42.92°, which suggests that there was incorporation of the larger Zn^{2+} ions in the MgO lattice.[3–5] The ZnO (110) peak for $x = 0$ is not noticeable above the background because undoped ZnO is strongly c-axis oriented and few ZnO (100) and (110) planes (which run parallel to the c-axis) diffract. The ZnO (110) peak has a lower intensity than the ZnO (100) peak, which itself is barely noticeable. But these peaks become more intense with Mg doping as the grains become more a-axis oriented.

Section S2. Pulsed Laser Deposited $Zn_{1-x}Mg_xO$

The AALD $Zn_{1-x}Mg_xO$ films were deposited in air at relatively low temperatures (150 °C), which is compatible with manufacturing on a commercial scale. For comparison, we also prepared $Zn_{1-x}Mg_xO$ films using a standard vacuum-based technique, pulsed laser deposition (PLD).

The PLD films were grown at 450 °C and made under a low oxygen partial pressure of 10 mTorr. A PLD/MBE-2300 system from PVD Products, Inc. was used for the deposition. A KrF excimer laser (248 nm wavelength, 400 mJ energy) with a pulse duration of 20 ns and repetition rate of 10 Hz was used for the ablation of the target. A ZnO target was used for depositing undoped ZnO. For depositing Mg-doped ZnO, the target was made by mixing together ZnO and MgO powders with the desired concentration, followed by grinding via mechanical milling. The mixture was then calcined in air and pressed at 40 MPa into 25 mm diameter discs. These were sintered at 900 °C.

The PLD films were highly crystalline and c-axis oriented (Figure S2a), and had bandgaps that corresponded with those of the AALD films with the same Mg doping (as seen from the excitonic peaks in Figure S2b and the onset of absorption in Figure S2c). The presence of band tails, evident in Figure S2b-d, is in agreement with the polycrystalline nature of the films, as observed by atomic force microscopy (Figure S3b and S3d), depsite the high temperature of deposition.

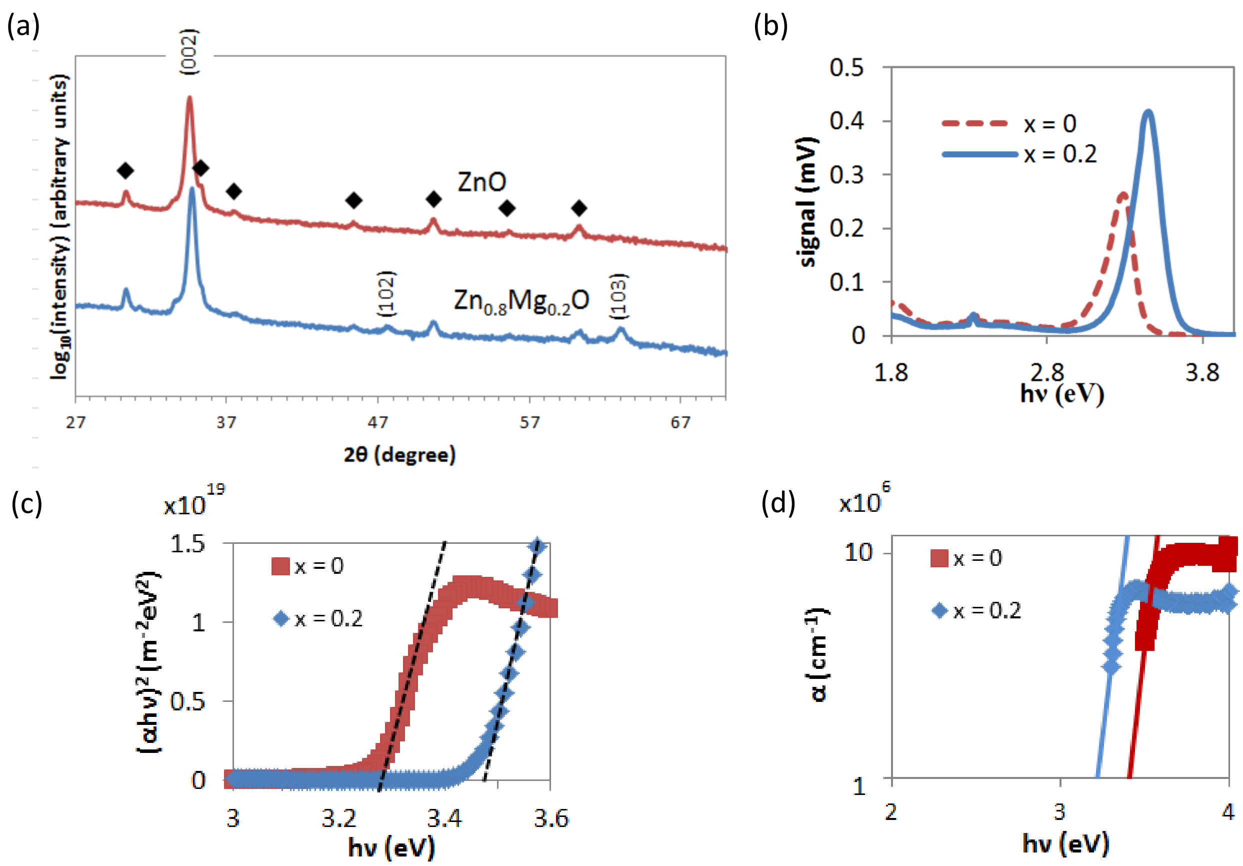

Figure S2. (a) X-ray Diffraction patterns of pulsed laser deposited (PLD) $Zn_{1-x}Mg_xO$ films on ITO/glass substrates, with the ITO peaks indicated by black diamonds and the ZnO peaks identified by their Miller indices. (b) Photoluminescence spectra of PLD ZnO and PLD $Zn_{0.8}Mg_{0.2}O$ films. (c) Tauc plots of undoped PLD ZnO and PLD $Zn_{0.8}Mg_{0.2}O$ films, and (d) the corresponding absorbance plots with the fitted Urbach models.

The CQDSCs showed an increase in the V_{OC} and efficiency with Mg doping, as expected, and the change in the J_{SC}s and *FF*s did not exceed the uncertainties in those quantities (Table S2).

Table S2. Comparison of the photovoltaic performance of $Zn_{1-x}Mg_xO$ – PbSe CQDSCs with PLD $Zn_{1-x}Mg_xO$.

Sample	V_{OC} (V)	J_{SC} ($mA.cm^{-2}$)	*FF* (%)	*PCE* (%)	R_{Shunt} ($\Omega.cm^2$)	R_{Series} ($\Omega.cm^2$)
PLD ZnO – PbSe	0.29±0.04	-9.3±0.5	31±1	0.8±0.2	50±9	22±1
PLD $Zn_{0.8}Mg_{0.2}O$ - PbSe	0.36±0.02	-9.8±0.3	32±1	1.1±0.1	63±5	23±1

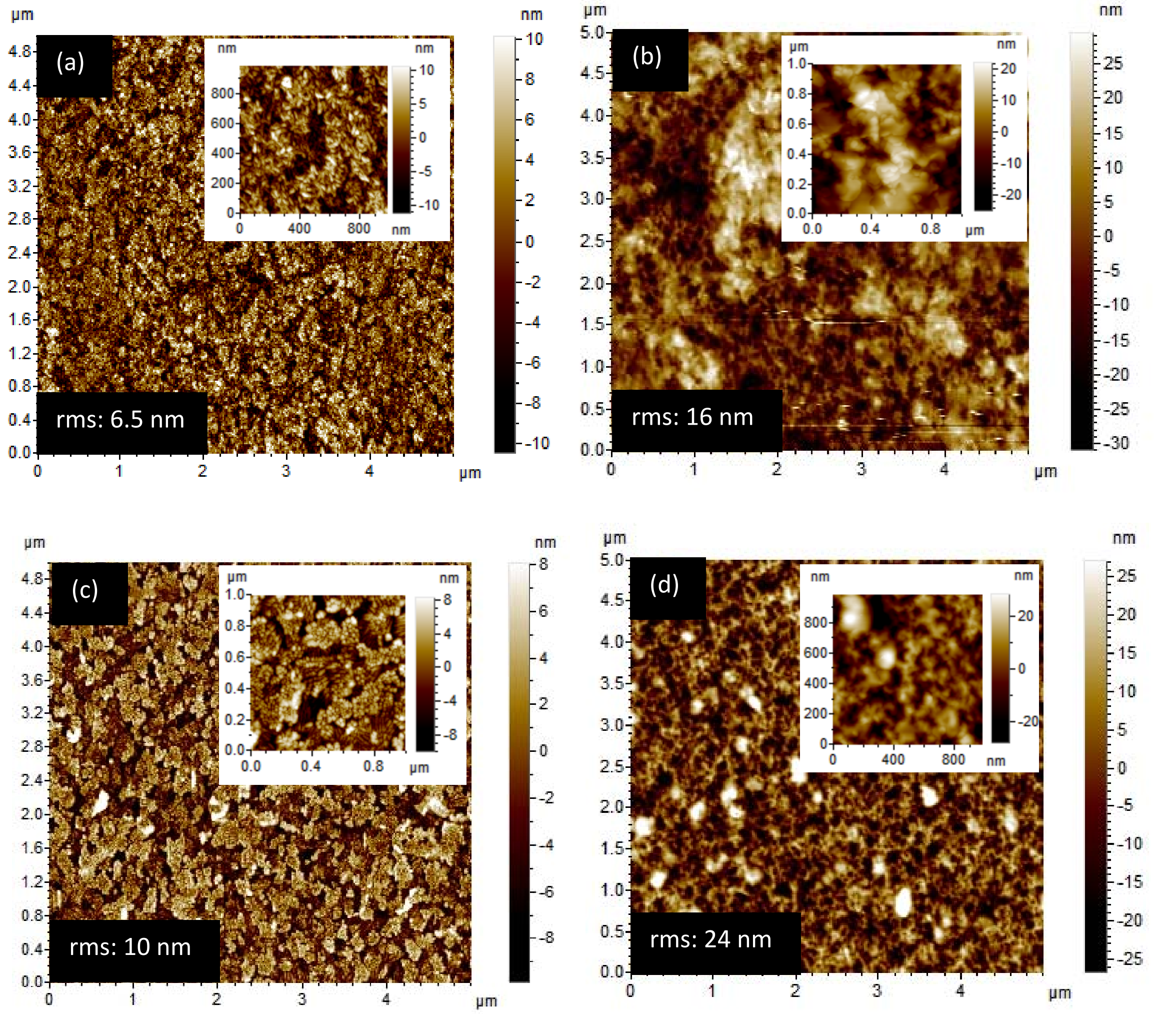

Figure S3. AFM topography images of (a) AALD ZnO (b) PLD ZnO, (c) ITO substrate, and (d) PLD $Zn_{0.8}Mg_{0.2}O$. The topography over a 1 µm x 1 µm area for each film is inset.

The PLD films were rougher than the AALD films, as can be seen from the topography images in Figure S3. The AALD film was highly conformal to the substrate (as seen by comparing Figure S3a with Figure S3c) and the roughness was mainly due to the ITO substrate. The high degree of smoothness would have led to the deposition of a uniform PbSe QD layer. By contrast, the PLD films had large vertical variations in thickness (~30 nm) that were significant compared with the 100 nm thick PbSe QD layer (Figure S3b and S3d). The PbSe QD layers deposited onto these PLD films would have been less uniform, which may have affected light absorption and charge transport within the film, resulting in the observed reduction in device perfromance as compared with the devices using AALD $Zn_{1-x}Mg_xO$. Other factors, such as $Zn_{1-x}Mg_xO$ carrier properties, compactness, transmittance and the influence of high temperature processing on the ITO may also have influenced the

performance of the devices using PLD $Zn_{1-x}Mg_xO$, but a detailed investigation is beyond the scope of this work.

Section S3. PbSe QD Bandgap Measurement

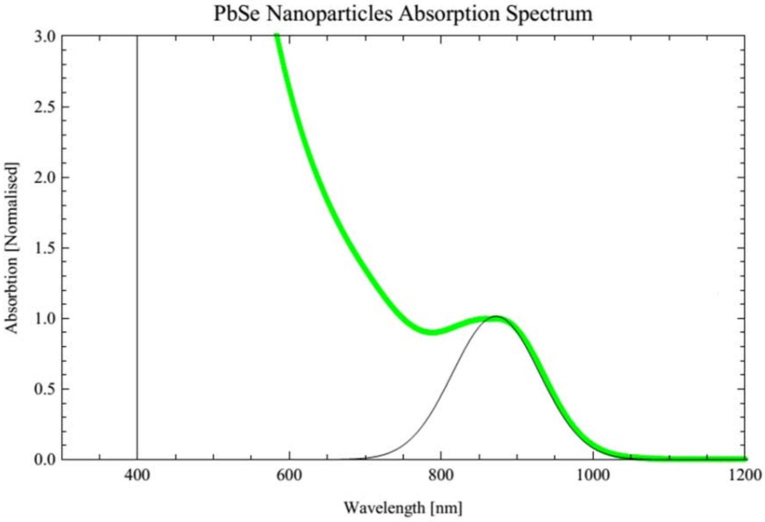

Figure S4. UV-visible spectroscopy measurement of the 1.43 eV band gap PbSe QDs used in this work. Previous measurements have indicated that the valence band position of our PbSe QDs remain at -5.1 eV, regardless of the bandgap.[6] Since the bandgap was measured as 1.43 eV, the conduction band position of these QDs would have been approximately -3.67 eV.

Section S4. Measurements of Sub-bandgap States by Absorption Measurements

Absorption measurements (photothermal deflection spectroscopy and UV-visible spectroscopy) were used to further examine the tail of sub-bandgap states in the $Zn_{1-x}Mg_xO$ films. Photothermal deflection spectroscopy (PDS) measurements were obtained for absorption below the bandgap by shining monochromatic light (Pump beam) onto the sample and heating it to create a thermal gradient and a refractive index gradient in the area around the sample surface. This refractive index gradient was enhanced by immersing the sample in an inert liquid called Fluorinert FC-72. A fixed wavelength (670 nm) CW laser beam (Probe beam) was passed through the thermal gradient and was deflected due to the refractive index gradient. This deflection was proportional to the absorbed light at that particular wavelength. These deflections were measured using a position sensing detector and lock-in detector. The PDS measurements were performed for $x = 0$ and $x = 0.42$ and combined in Figures S5a-c with above-bandgap absorbance values determined using UV-visible spectroscopy.

The PDS measurements show absorption due to defect states within the bandgap, in addition to the tail of sub-bandgap states. To deconvolute these contributions, we fitted the Urbach model to the absorption front. The Urbach model is given by Eq. S1:[7]

$$\alpha(h\upsilon) = \alpha_0 e^{\frac{h\upsilon - E_g}{E_U}} \qquad \text{(S1)}$$

where E_U is the Urbach energy, α the absorption coefficient, E_g the bandgap, $h\nu$ the photon energy and α_0 a constant.[7]

The Urbach fit was applied to the absorption front in the log-linear absorption plots (Figure S5a and S5b). A comparison of the fitted Urbach models to the absorbance data is given in Figure S5a-c, showing that there is a good fit. If we compare the Urbach models themselves to observe the absorption only due to the tail of sub-bandgap states, we can see that as the conduction band was raised, the energy required for sub-bandgap absorption also increased (Figure S5d). This is in agreement with the photoluminescence spectra data in Figure 3b and suggests that the available electron acceptor levels due to any sub-bandgap states below the $Zn_{1-x}Mg_xO$ conduction band were shifted to higher energies as the conduction band was shifted upwards by Mg doping.

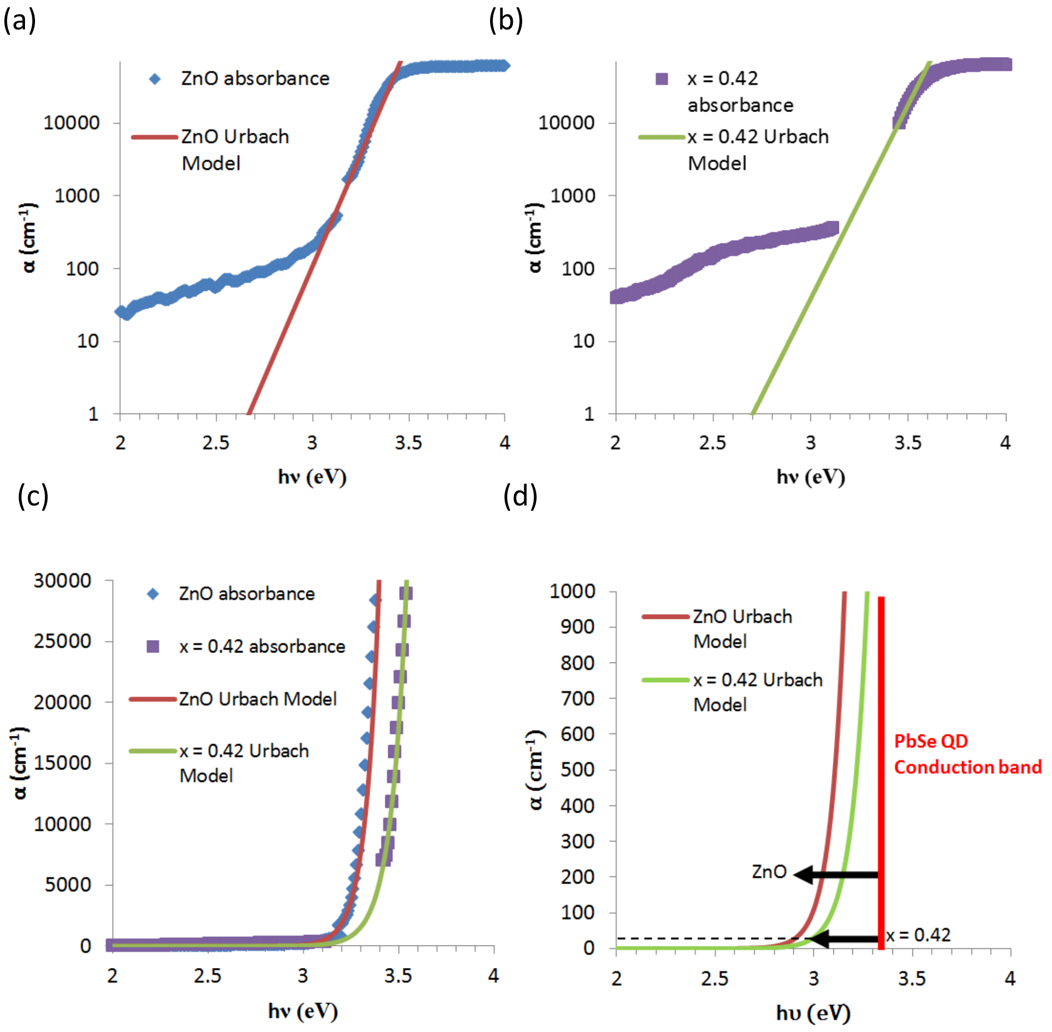

Figure S5. Logarithmic-linear plots of absorption coefficient (α) vs. photon energy (hv) to show the defect states and fit of the Urbach models for (a) ZnO and (b) $Zn_{0.56}Mg_{0.42}O$ films. There are missing data points because only data above the bandgap in the transmission data was used (to avoid interference fringes), whereas the PDS measurements could only be performed below 3.2 eV. (c) Comparison of the absorbance data and Urbach models on linear-linear axes. (d) Comparison of the Urbach models only of both films to the approximate PbSe conduction band level (in red) showing the shift in the tail of the density of sub-bandgap states.

Section S5. Hall-Effect Measurements of AALD $Zn_{1-x}Mg_xO$ Films

The carrier concentration and mobility of the $Zn_{1-x}Mg_xO$ films were measured using the Van der Pauw method in a Hall effect rig. The sheet resistance was first measured with a Keithley 6220 current source and 2182A Nanovoltmeter. A 1 T magnetic field was then applied and the current source and the Nanovoltmeter used to measure the Hall voltage, from which the carrier concentration was calculated. The mobility was calculated from the carrier concentration and resistivity.[1,8]

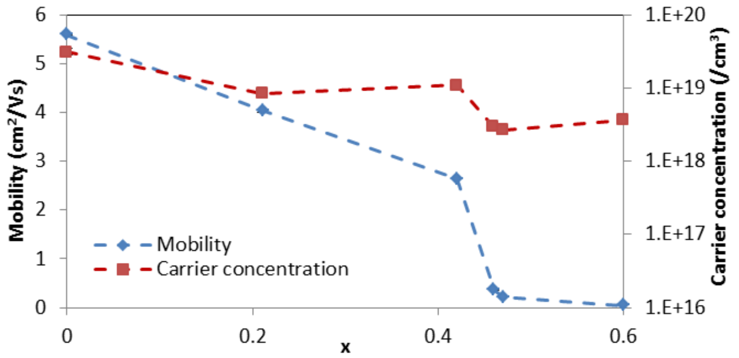

Figure S6. The change in mobility and carrier concentration of AALD $Zn_{1-x}Mg_xO$ films with Mg content (x) measured under laboratory lighting.

The decrease in the carrier concentration found (Figure S6) may have been due to a change in the formation of point defects in the ZnO with Mg doping.[9] The decrease in the carrier concentration observed here was smaller than that previously reported for N-doped ZnO.[10] This is because N has a higher valency than O, meaning that N would remove more free electrons than O, leading to a decrease in the carrier concentration. Mg, however, has the same valency as Zn and should not donate fewer free electrons to the lattice than Zn.

In Figure 2b in the manuscript, there is a decrease in J_{SC} for $x > 0.21$. As discussed in the manuscript, this was due to a significant extent to the reduction in the accessible density of ZnO band-tail states as the conduction band was raised. The J_{SC}s reduction can also be attributed in part to a reduced electron mobility in the $Zn_{1-x}Mg_xO$ upon doping (Figure S6), which is expected to arise from an increase in the effective electron mass and alloy disorder scattering.[11]

Section S6. Photointensity measurements

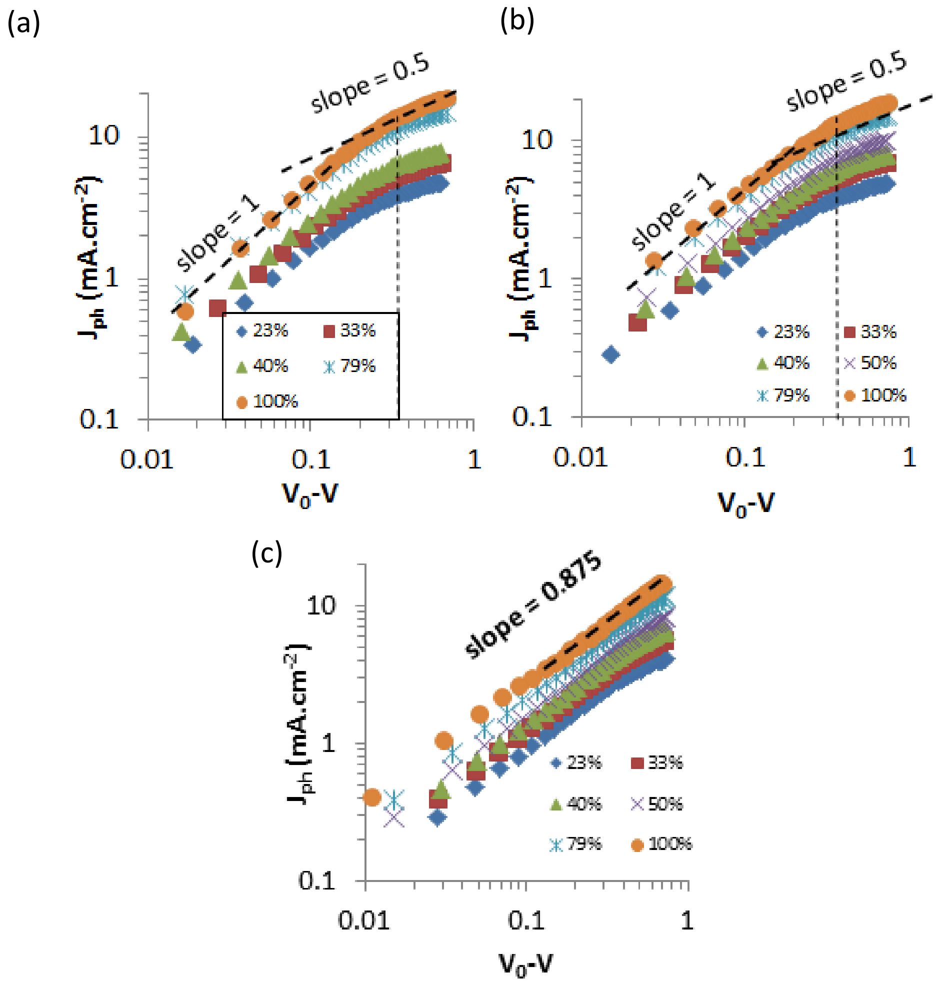

Figure S7. Photocurrent (J_{ph}) vs. effective applied bias (V_0-V) for (a) x = 0, (b) x = 0.21 and (c) x = 0.42. The legends for each plot indicate the percentage of the light intensity of 1 sun (100 mW.cm^{-2} under AM 1.5G illumination).

From Figure S7a and S7b, it can be seen that an effective applied bias ($V_0 - V$) of 0.35 V occurs in the square-root regime (slope = 0.5 in a log-log plot) for the devices with x = 0 and x = 0.21.

The photocurrent vs. effective applied bias log-log plot for the devices with x = 0.42 (Figure S7c) was unconventional as it did not have a region where the slope was unity or 0.5. Rather, the slope was 0.875. This could be because this device does not follow the assumptions made by Goodman *et al.* when they derived the governing equations for current extraction. For example, the device with x = 0.42 had more mid-bandgap defect states than lower doping levels, whereas Goodman *et al.* assumed that there were no defect states present.[12] As a result, it was not possible to use this method to detect if there was a space-charge region in the

device with $x = 0.42$. However, measurements of the fill factor vs. light intensity and the white-light bias EQE for $x = 0.42$ (Figure 5b and Figure S8) indicate a reduction in the efficiency of charge collection from the quantum dots, which is consistent with the presence of a space-charge region in the quantum dot layer, and supports the idea that the metal oxide has an active conduction band tail.

White-light biased EQE measurements were performed using a 250 W tungsten light source (chopped using a Thorlabs MC2000 mechanical chopper) coupled to an Oriel Cornerstone 130 monochromator. The white-light bias illumination was provided using a white LED array and calibrated using a silicon photodiode and solar simulator. To differentiate the signal due to the monochromatic illumination (which had an intensity of less than 1 $mW.cm^{-2}$) from that due to the white light bias, the current from the device was passed to a Stanford Research SR 570 current preamplifier to subtract the constant background before being fed into a Femto LIA-MVD-200 lock-in amplifier.

Figure S8 shows that with no white-light background illumination (WLB), the EQE increased monotonically with doping, even under an applied bias. The generation rate of the photogenerated carriers was suitably small, such that charge transfer was efficient, even in the device with $x = 0.42$. The reduction in interfacial recombination with Mg doping therefore resulted in a slightly higher EQE for the devices with Mg-doped ZnO. With WLB (1 sun), the cell with the highest doping ($x = 0.42$) showed the lowest EQE due to limited electron transfer from the PbSe to the smaller density of accepting states in the oxide.

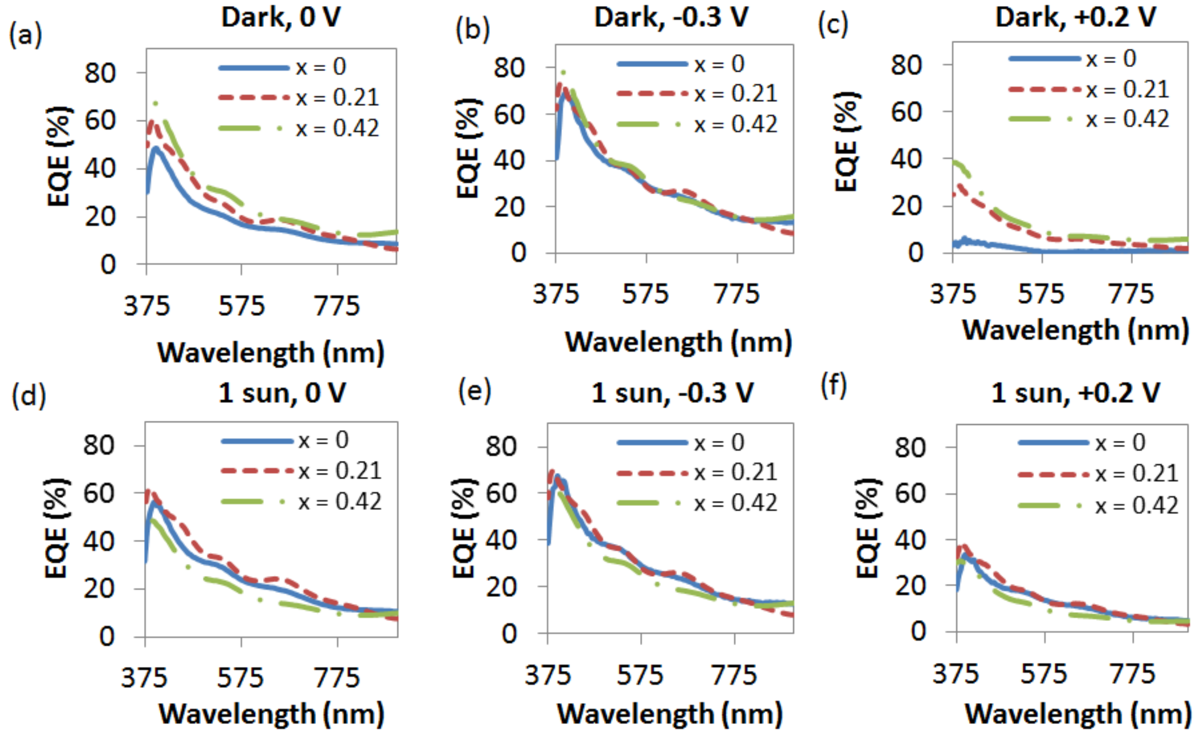

Figure S8. EQE data obtained at different applied biases and white light background illumination. (a) 0 V bias, no illumination, (b) -0.3 V bias, no illumination, (c) +0.2 V bias, no illumination. (d) 0 V bias, 1-sun illumination, (e) -0.3 V bias, 1-sun illumination, (f) +0.2 V bias, 1-sun illumination.

References

[1] R. L. Z. Hoye, D. Muñoz-Rojas, D. C. Iza, K. P. Musselman, J. L. MacManus-Driscoll, *Sol. Energy Mater. Sol. Cells* **2013**, *116*, 197.

[2] D. Muñoz-Rojas, H. Sun, D. C. Iza, J. Weickert, L. Chen, H. Wang, L. Schmidt-Mende, J. L. MacManus-Driscoll, *Prog. Photovoltaics Res. Appl.* **2013**, *21*, 393.

[3] D. A. Fletcher, R. F. McMeeking, D. Parkin, *J. Chem. Inf. Comput. Sci.* **1996**, *36*, 746.

[4] A. Kern, R. Doetzer, W. Eysel, *MgO - ICDD Grant-in-Aid*, Heidelberg, Germany, **1993**.

[5] V. Etacheri, R. Roshan, V. Kumar, *ACS Appl. Mater. Interfaces* **2012**, *4*, 2717.

[6] B. Ehrler, B. J. Walker, M. L. Böhm, M. W. B. Wilson, Y. Vaynzof, R. H. Friend, N. C. Greenham, *Nat. Commun.* **2012**, *3*, 1019.

[7] A. Meeder, D. F. Marrón, A. Rumberg, M. C. Lux-Steiner, V. Chu, J. P. Conde, *J. Appl. Phys.* **2002**, *92*, 3016.

[8] D. Muñoz-Rojas, M. Jordan, C. Yeoh, A. T. Marin, A. Kursumovic, L. A. Dunlop, D. C. Iza, A. Chen, H. Wang, J. L. MacManus Driscoll, *AIP Adv.* **2012**, *2*, 042179.

[9] Y. Ke, J. Berry, P. Parilla, A. Zakutayev, R. O'Hayre, D. Ginley, *Thin Solid Films* **2012**, *520*, 3697.

[10] B. Ehrler, K. P. Musselman, M. L. Böhm, F. S. F. Morgenstern, Y. Vaynzof, B. J. Walker, J. L. MacManus-Driscoll, N. C. Greenham, *ACS Nano* **2013**, *7*, 4210.

[11] J. Piris, N. Kopidakis, D. C. Olson, S. E. Shaheen, D. S. Ginley, G. Rumbles, *Adv. Funct. Mater.* **2007**, *17*, 3849.

[12] A. M. Goodman, A. Rose, *J. Appl. Phys.* **1971**, *42*, 2823.

13
